# Exploring the IRE1 interactome: From canonical signaling functions to unexpected roles

**DOI:** 10.1016/j.jbc.2024.107169

**Published:** 2024-03-15

**Authors:** Simon Le Goupil, Hadrien Laprade, Marc Aubry, Eric Chevet

**Affiliations:** 1 INSERM U1242, University of Rennes, Rennes, France; 2 Centre de Lutte contre le cancer Eugène Marquis, Rennes, France

**Keywords:** endoplasmic reticulum, protein homeostasis, unfolded protein response, IRE1, signaling, stress, interactome

## Abstract

The unfolded protein response is a mechanism aiming at restoring endoplasmic reticulum (ER) homeostasis and is likely involved in other adaptive pathways. The unfolded protein response is transduced by three proteins acting as sensors and triggering downstream signaling pathways. Among them, inositol-requiring enzyme 1 alpha (IRE1α) (referred to as IRE1 hereafter), an endoplasmic reticulum–resident type I transmembrane protein, exerts its function through both kinase and endoribonuclease activities, resulting in both X-box binding protein 1 mRNA splicing and RNA degradation (regulated ire1 dependent decay). An increasing number of studies have reported protein–protein interactions as regulators of these signaling mechanisms, and additionally, driving other noncanonical functions. In this review, we deliver evolutive and structural insights on IRE1 and further describe how this protein interaction network (interactome) regulates IRE1 signaling abilities or mediates other cellular processes through catalytic-independent mechanisms. Moreover, we focus on newly discovered targets of IRE1 kinase activity and discuss potentially novel IRE1 functions based on the nature of the interactome, thereby identifying new fields to explore regarding this protein’s biological roles.

## Endoplasmic reticulum stress and the unfolded protein response

The endoplasmic reticulum (ER) is one of the largest organelles in eukaryotic cells. It was observed for the first time at the end of the 19th century by Charles Garnier and named “ergastoplasm” (1) then defined as ER in 1945 by Porter, Claude, and Fullam (2). Beyond its functions in the regulation of calcium, cells’ redox status and lipid homeostasis, the ER has a predominant role in protein synthesis, productive folding, and trafficking (3, 4, 5, 6). Indeed, about one-third of the proteome passes through the ER to acquire proper folding before reaching its final destination (7). The ER lumen exhibits a calcium-rich, oxidative environment populated by ER-resident enzymes and foldases (*e.g.*, protein disulfide isomerases (PDIs), chaperones, N-linked glycosylation machinery), which ensure proper protein folding (8, 9). In addition, a protein quality control system is in charge of either retaining improperly folded proteins in the ER to allow further folding cycles or to promote the degradation of terminally misfolded proteins through ER-associated degradation (ERAD) (10). This process is critical to ensure ER homeostasis, integrating protein synthesis rates, folding, and degradation/export through the secretory pathway (6). Under some circumstances, ER homeostasis can be disrupted when protein folding demand in the ER exceeds the ER capacity to handle productive protein folding. This leads to an accumulation of improperly folded proteins in this compartment, a situation known as ER stress (11). To cope with ER stress, organisms/cells have evolved a strategy termed the unfolded protein response (UPR). The UPR is a signaling pathway triggered upon ER stress mainly to restore ER homeostasis (adaptive UPR). However, if this process fails, the UPR will engage cells on a death path (terminal UPR) (12, 13). Over the years, many studies showed the involvement of UPR signaling in a wide range of diseases such as cancers, metabolic, or degenerative disorders (14, 15).

**Figure 1:**
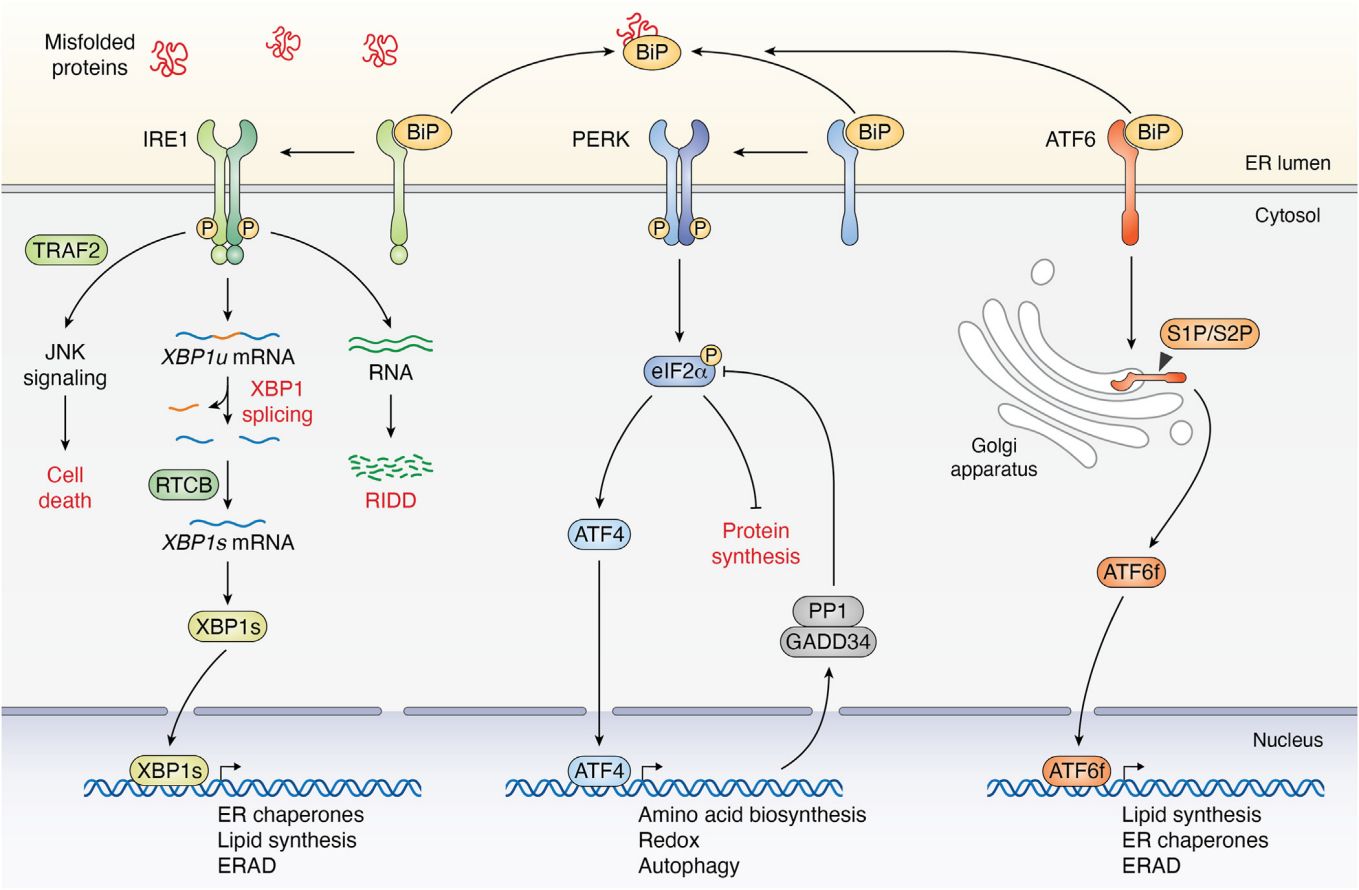
**The unfolded protein response.** Schematic representation of the IRE1, PERK, and ATF6 branches of the UPR. ATF6, activating transcription factor 6; IRE1, inositol-requiring enzyme 1; PERK, PKR-like endoplasmic reticulum kinase.

The UPR is transduced by three ER-resident transmembrane sensors named respectively the PKR-like endoplasmic reticulum kinase (PERK), the activating transcription factor 6 (ATF6), and the inositol-requiring enzyme 1 (IRE1). PERK is a type 1 transmembrane protein with a luminal sensing domain and a cytosolic kinase catalytic domain. Activated PERK enables the phosphorylation of the eukaryotic translation initiation factor 2A (eIF2α), thereby decreasing overall mRNA translation and therefore the ER protein load (16). However, phosphorylated eIF2α allows bypassing the reading of upstream ORF on translated mRNA by ribosomes to activate the selective synthesis of several proteins, including the ATF4 transcription factor (17). ATF4 targets the transcription of genes whose products are involved in redox homeostasis, amino acid metabolism, or autophagy (18, 19) (Fig. 1). The ATF4-dependent transcription network is also contributing to a negative feedback loop allowing translation to resume (20). Indeed, ATF4 controls the expression of the phosphatase subunit GADD34 which associates with the protein phosphatase 1 catalytic subunit to dephosphorylate eIF2α hence restoring translation (21). Upon accumulation of improperly folded proteins in the ER lumen, the type II ER transmembrane protein ATF6 is translocated in the Golgi apparatus through coat protein complex II transport vesicles, where it is cleaved by the site 1/2 proteases (S1P and S2P) leading to the release of ATF6 cytosolic domain (CD) (ATF6f) in the cytosol (22). ATF6f is a leucine zipper (bZIP) motif containing transcription factor that mostly controls the expression of genes coding for ER chaperones/foldases, thereby leading to an increase in productive protein folding in the ER (23, 24) (Fig. 1).

The most evolutionary conserved stress transducer of the UPR is IRE1, discovered in yeast and mammals in the 90s (25, 26). IRE1 is an ER-resident type 1 transmembrane protein that comprises both BiP-binding (binding-immunoglobulin protein) and misfolded protein binding domain in the ER lumen as well as kinase and endoribonuclease catalytic domains in the cytosol. Upon ER stress, IRE1 oligomerizes and transautophosphorylates, triggering two outcomes managed by the RNase domain (25, 27). First, the nonconventional splicing of X-box binding protein 1 (XBP1) mRNA, where IRE1 removes a 26-nt intron, with further ligation of the remaining 5′ and 3′ fragments by the tRNA ligase RtcB to form the spliced XBP1 (XBP1s) mRNA (28, 29, 30). This splicing event causes a frameshift in the XBP1s transcript, leading to translation of a protein containing a transcription activator domain in the C-terminal end. Hence XBP1s acts as a transcription factor regulating the expression of enzymes involved in lipid biogenesis, of ER chaperones, and ERAD components (31, 32, 33, 34). Second, the RNase activity termed as regulated IRE1dependent decay (RIDD) is responsible for cleaving a pool of RNAs (mRNA, rRNA, miRNA), aiming at decreasing translation or at inducing novel cellular programs (35, 36) (Fig. 1). Importantly, RIDD specificity still remains unclear and might be cell specific. To date, a consensus sequence was identified for RIDD substrates (CUGCAG) within a stem–loop motif (37), but a recent study unveiled targeting of RNAs lacking those criteria and termed this activity as RIDD-lacking endomotif (38). In addition to their signaling, the three UPR sensors exerts a crosstalk between each other as shown by the upregulation of XBP1 expression by both ATF6f and ATF4 (39, 40) or the competitive role of PERK and IRE1 regarding expression of the death receptor 5 (41). Some hypotheses propose a hierarchy among the UPR sensors in human, suggesting that low ER stress is solely resolved by the ATF6 branch, while mild or high ER stress additionally requires IRE1 and PERK branches. Moreover, upon persistent ER stress, it is thought that IRE1 signaling declines in the benefit of PERK, to mediate CHOP-induced apoptosis (42). Numerous studies also point toward scaffolding functions of IRE1 either to regulate its signaling or to mediate other cellular processes (translation, cytoskeleton …). In this review, we propose an overview of IRE1 biochemistry and describe the known IRE1 protein-–protein interactions (PPis) (Table 1) and their biological outputs.

**Table 1:** List of described IRE1 interactors

Biological functions	Protein ID	Protein full name	Reference
IRE1 activation	Misfolded proteins	Misfolded proteins	(85, 86, 87)
	AGR2∗	Anterior gradient protein 2	(51, 95)
	BIP	Endoplasmic reticulum chaperone BiP	(88)
	ERDJ4	DnaJ homolog subfamily B member 9	(88)
	HSP47	Serpin family H member 1	(96)
	PDIA1	Protein disulfide isomerase A1	(99)
	PDIA6	Protein disulfide isomerase A6	(98)
IRE1 Oligomerization	c-ABL	Tyrosine-protein kinase ABL1	(104, 105)
	NMIIB	Nonmuscle myosin heavy chain IIB	(106)
	RAC	Ribosome-associated complex	(103)
	Sec61β	Protein transport protein Sec61 subunit beta	(101, 102)
	Sec63	Translocation protein Sec63 homolog	(101, 102)
IRE1 RNase activity	BAK	Bcl-2 homologous antagonist/killer	(109)
	BAX	Apoptosis regulator BAX	(109)
	BI-1	BAX inhibitor 1	(112, 113)
	BIM	Bcl-2–like protein 11	(110)
	c-ABL	Tyrosine-protein kinase ABL1	(104)
	NCK	Cytoplasmic protein NCK1	(114)
	PUMA	Bcl-2–binding component 3	(110)
	RNH1	Ribonuclease inhibitor	(111)
IRE1 Phospho regulation	AMPK	AMP-activated protein kinase	(118, 119)
	Dcr2	Phosphatase DCR2 (*S. cerevisiae*)	(116)
	ERK	Extracellular signal–regulated kinase	(120)
	PKA	Protein kinase A	(122)
	PP2A	Protein phosphatase 2A	(118)
	PP2Ce	Protein phosphatase 1L	(117)
	Ptcp2	Serine/threonine phosphatase of type 2C (*S. cerevisiae*)	(26)
	PTP1B	Tyrosine-protein phosphatase nonreceptor type 1	(105, 121)
	RACK1	Receptor for activated C-kinase 1	(118)
	RPAP2	RNA polymerase II–associated protein 2	(41)
	SCP3	Small carboxy-terminal domain phosphatase 3	(120)
PTM and IRE1 degradation	BIM	Bcl-2–like protein 11	(110)
	CHIP	E3 ubiquitin-protein ligase CHIP	(129)
	M50	M50 viral protein	(128)
	MITOL	E3 ubiquitin-protein ligase MARCHF5	(131)
	OS9	OS9 endoplasmic reticulum lectin	(127)
	RNF13	E3 ubiquitin-protein ligase RNF13	(130)
	SEL1L	Protein sel-1 homolog 1	(126, 127)
	SYNV1	E3 ubiquitin-protein ligase synoviolin	(126, 127)
Apoptosis	AIP1	ASK1-interacting protein-1	(137)
	ASK1	Apoptotic signaling kinase 1	(135, 136)
	JNK	Mitogen-activated protein kinase 8	(135)
	MITOL	E3 ubiquitin-protein ligase MARCHF5	(131)
	TRAF2	TNF receptor–associated factor 2	(135)
Cytoskeleton	CORO1	Coronin	(111)
	FLNA	Filamin A	(139)
	PKCα	Protein kinase C alpha type	(139)
	TUBA	Tubulin A	(111)
	TUBB	Tubulin B	(111)
SRP and ribosome	RPLs	Ribosome protein large subunits	(143)
	SRPs	Signal recognition particles	(143)
Ca^2+^ homeostasis	INSP3R	Inositol 1,4,5-trisphosphate receptor type 3	(148)
	STIM1	Stromal interaction molecule 1	(149)
IRE1 Kinase target	AMPK	AMP-activated protein kinase	(119)
	BCL2	Apoptosis regulator Bcl-2	(119)
	FMRP	Fragile X messenger ribonucleoprotein 1	(150)
	IKK	IκBα kinase	(152)
	PUM1	Pumilio homolog 1	(151)
	Pumilio	Pumilio (*Drosophilia melanogaster*)	(151)
	SPL	Sphingosine 1-phosphate lyase	(153)

IRE1, inositol-requiring enzyme 1; PTM, posttranslational modification.

(∗) indicates IRE1β specific interactions.

## IRE1 across evolution

### IRE1α and IRE1β paralogs

The evolution of the IRE1 gene might have encountered a duplication event, as, unlike in *Saccharomyces cerevisiae*, two IRE1 paralogs exist in mammals, namely IRE1α and IRE1β, respectively encoded by two genes: ERN1 and ERN2, respectively localized on 17q23.3 and 16p12.2 chromosomal regions and displaying 39% sequence identity (43). Both proteins conserved their structural domains. Whereas IRE1α is ubiquitously expressed, IRE1β is preferentially found in mucosal epithelia (44, 45). Regarding their functions, both IRE1α and IREβ exerts RIDD activity and IRE1β was found to inhibit mRNA translation by degrading rRNA (43). However, while IRE1α-mediated XBP1 mRNA splicing has been largely described, it remains unclear whether IRE1β is capable of such activity in a physiological setting. Due to nonconserved amino acids in the kinase domain, IRE1β has impaired phosphorylation and reduced oligomerization abilities (46, 47, 48). Even so, few studies were able to observe a slight XBP1 splicing activity for IRE1β in *in vitro* splicing experiments and in overexpressing cells (49, 50, 51). In the latter case, the splicing activity is dependent on cell type and appeared to be reduced in mucosal cell line where IRE1β is usually expressed (51). Concomitantly, IRE1β was found to act as a negative regulator of IRE1α, as its overexpression is able to decrease IRE1α-dependent XBP1 splicing (47). Indeed, IRE1β conserved a capacity to oligomerize with IRE1α and by doing so, acts as a dominant negative by preventing IRE1α functions (47). In this regard, IRE1β is thought to attenuate ER stress upon inflammation induced diseases in mucosal epithelium (44). For the continuation of this review, we will mainly consider IRE1α (and refer to it as IRE1 unless specified).

### IRE1 across evolution

**Figure 2:**
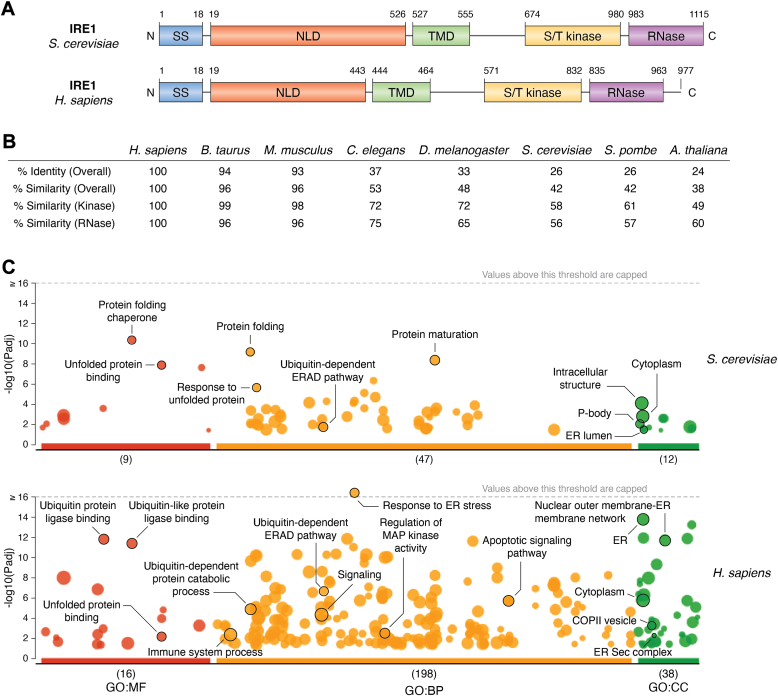
**Evolutive insights on IRE1.***A*, schematic representation of yeast (*above*) and human (*below*) IRE1 sequence displaying the signal peptide (SP) with the three mains domains: luminal (NLD), a transmembrane domain, and the cytosolic domain exerting kinase and RNase activities. *B*, comparison of IRE1 protein sequence identity and similarity in different species using BLAST. *C*, GO Manhattan plot using g:Profiler of yeast (*up*) and human (*bottom*) IRE1 interactome from curated databases (APID, String and Biogrid). *Black arrows* highlight terms of interest, and values in *brackets* represent the number of enriched terms in each Gene Ontology. IRE1, inositol-requiring enzyme 1; NLD, N-terminal luminal domain.

IRE1 can be divided into three main domains: an N-terminal luminal domain (NLD), a transmembrane domain (TMD), and a CD, the latter comprising both kinase and RNase catalytic activities. These domains are all conserved in metazoan, fungi, and plant orthologs, as shown by the yeast *versus* human IRE1 comparison (Fig. 2*A*) (52). However, compared to human IRE1, the amino acid sequence differs across the reigns. Indeed, while the BLAST alignment of protein sequences reveals a high degree of conservation in mammals (>90% identity), a low IRE1 sequence conservation is found between human and the other species (approximatively 30% identity according to species) (Fig. 2*B*) (53). This trend is comparable with the overall similarity of the sequences (>90% in mammals while approximatively 50% in other eukaryotes and <40% in plants) (Fig. 2*B*). This suggests that both IRE1 kinase and RNase domains share common features and functions across species despite the changes in sequences and implies that the luminal domain diverges more and may show different characteristics which may be linked to (i) IRE1 activation mode in different species and/or (ii) the ability of IRE1 luminal domain to directly and selectively bind to specific motifs which may differ between species. However, IRE1 activity presents significant differences in yeast species. In *S. cerevisiae*, IRE1 can splice HAC1 mRNA (XBP1 functional homolog in yeast) together with the *S. cerevisiae* Rlg1p ligase (RtcB homolog in yeast) (27, 54) and to a much lesser extent RIDD (55). Whereas in *S. pombe*, IRE1 only degrades RNA through RIDD since further analysis found that *S. pombe* lacks HAC1 (or orthologs) (55, 56). Therefore, one can hypothesize that all eukaryotes may express an IRE1 capable of both splicing and RIDD activities (with different selectivity) and that the occurrence of splicing does not depend on the IRE1 RNase domain but on the expression of the target gene. This suggests an evolutive trait of the IRE1 pathway in managing ER stress, by modifying signaling characteristics and likely to expand its biological functions during evolution. This correlates also with the diversification of the UPR, with the appearance of the PERK and ATF6 arms (57) in higher order organisms (metazoans and chordates).

### Comparison of IRE1 interactomes across evolution

To analyze the diversity of IRE1 PPis across evolution, we collected IRE1 interactome data from *S*. *cerevisiae* and *Homo sapiens*, the most documented species on public repositories (agile protein interactomes dataserver, String and Biogrid) (58, 59, 60). Interestingly, agile protein interactomes dataserver, the most complete database reports 300,000 interactions among the 5800 proteins studied in yeast while in human, 700,000 interactions are distributed among 14,500 proteins. Regarding IRE1, analysis of those repositories documents a total of 101 interactions for human against 121 for yeast. Gene Ontology analysis carried out through g:Profiler revealed a different pattern of enrichment between human and yeast IRE1 human interactome. Indeed, every ontology (molecular function, biological process, and cellular component) displays much more terms in human than in yeast (Fig. 2*C*) (61). Regarding the content of these ontologies, while in yeast IRE1 interactors are mainly linked to stress-related processes such as protein folding or ERAD, human IRE1 interactors are connected with many more diverse processes in addition to the UPR, from signaling pathways (with the mitogen-activated protein kinase (MAPK) signaling), trafficking (coat protein complex II) to apoptosis or ubiquitination (Fig. 2*C*). We analyzed the number of terms enriched between the interactomes of a significant number of *H. sapiens* and *S. cerevisiae* orthologs and found that the richer and denser functional enrichment observed in the human IRE1 interactome is significantly different (*p* value = 0.0014, not shown) than the mean of interactome differences of other orthologs. Altogether, it suggests that IRE1 is part of a specific subset of proteins, which gains new functions across evolution through their protein interaction network.

## IRE1 structural insights

### IRE1 luminal domain

**Figure 3:**
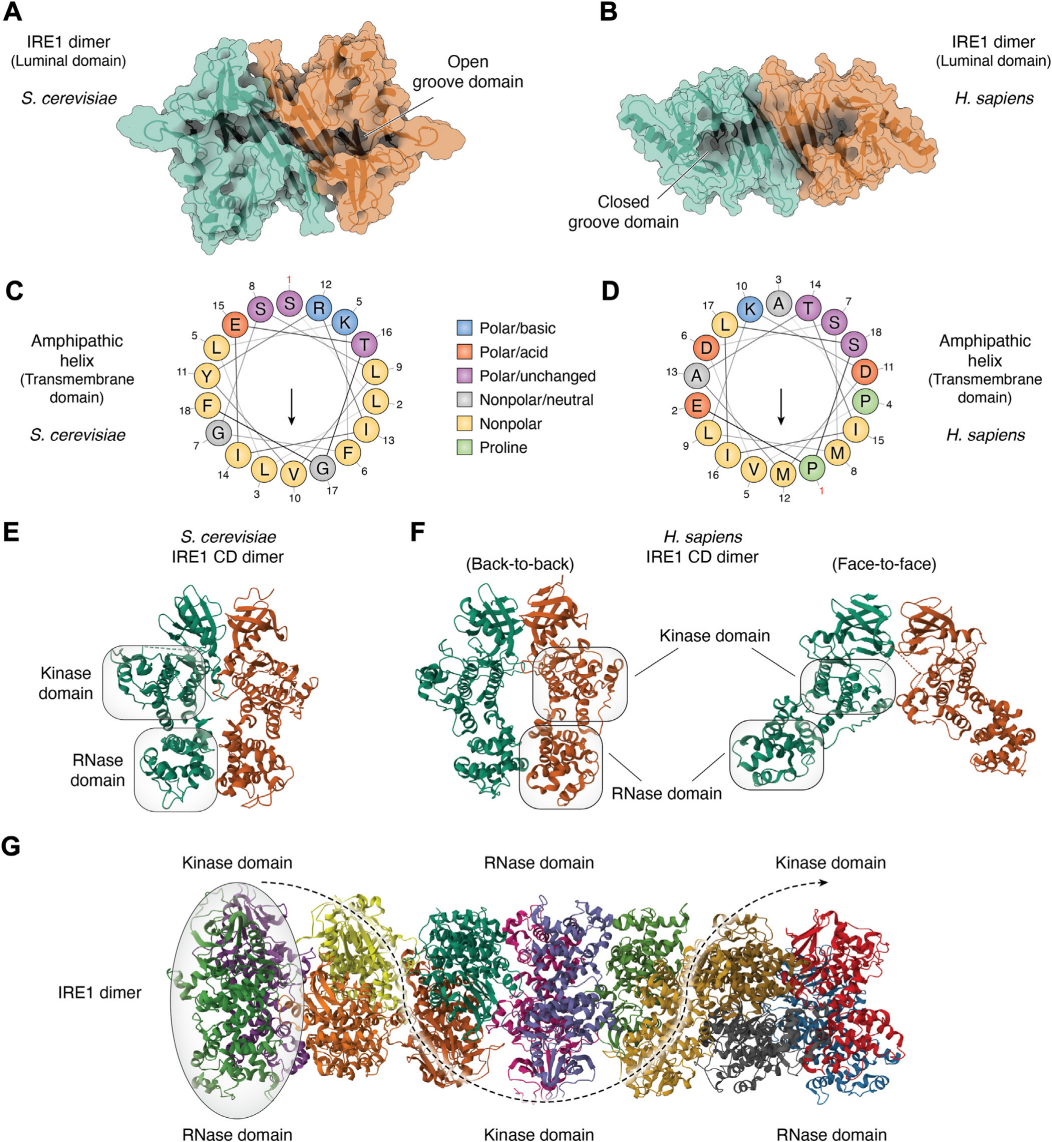
**Comparative structure of IRE1 in *Homo sapiens* and *Saccharomyces cerevisiae*.** Dimer structures of luminal domain of (*A*) yeast (PDB: 2BE1) and (*B*) human (PDB: 2HZ6) IRE1. Each color represents a monomer. Helical wheel schematic representation of IRE1 yeast (*C*) and (*D*) human amphipathic helix from the transmembrane domain. The *arrow* represents the hydrophobic moment. *E*, crystal structure of dimeric IRE1 cytosolic domain of yeast (PDB: 2RIO). *F*, crystal structures of human dimeric IRE1 cytosolic domain with back-to-back (PDB: 6W3C, *left*) and face-to-face (PDB: 3P23, *right*) conformations. *G*, crystal structure of yeast IRE1 cytosolic oligomer (PDB: 3FBV). Each color represents a monomer, and the *black arrow* illustrates the progressive 52° rotation between each dimer. IRE1, inositol-requiring enzyme 1.

A first crystal structure of the conserved core region of IRE1 NLD was obtained in *S. cerevisiae* in 2005 (62). It shows a dimer harboring a putative protein/protein interface (Fig. 3*A*). This structure describes that the IRE1 NLD is able to dimerize through both sides of the NLD and suggests that IRE1 could form high order oligomers necessary for its activation. This crystalized dimer also shows a “groove” structure between the two monomers similar to the major histocompatibility peptide-binding complexes (MHCs) (62). Interestingly, mutations in the NLD contact sites were shown to abolish IRE1 activation (63). In comparison, human IRE1 NLD dimer structure, obtained by crystallography, reveals a triangle shape of each monomer, composed of three β-sheets that appear different from the yeast NLD. This structural difference could be explained by the length of this domain in both species (Fig. 2*A*) and by the previously discussed poor conservation of the sequence as shown by the amino acid percentage identity between human and yeast (Fig. 2*B*). Upon dimerization, one face of a triangle interacts with another similar structure in an antiparallel manner, creating an MHC-like motif (Fig. 3*B*). This motif presents an interface able to form hydrophobic and hydrogen bonds, the last especially through the K121 amino acid (64). The following MHC-like structure is comparable to that of yeast, but the closed groove suggests a different peptide binding capacity in sensing unfolded protein (64). Of note, cancer-associated somatic mutations in the luminal domain of human IRE1 were reported to affect IRE1 signaling (65).

### IRE1 transmembrane domain

The IRE1 TMD consists in a single amino acid stretch exhibiting a juxta-membrane amphipathic helix (AH) containing polar residues facing the lumen and a transmembrane helix (66) (Fig. 3, *C* and *D*). Interestingly, analysis of the TMD from different species revealed a conservation of these AH and transmembrane helix from mammals to plants (66). In yeast, a study identified a link between IRE1 activation and lipid sensing properties regulated by IRE1 AH (66). It was found that the AH could sense lipid composition of the ER membrane and that mutation in this domain led to impaired HAC1 mRNA splicing (66). They further discussed a model in which IRE1 AH could locally compress the membrane, allowing stable formation of oligomers (66). Another study showed that lipid unbalance induces UPR and that IRE1 counteracts membrane stress-induced cell death by reprogramming protein homeostasis and maintains membrane functions without affecting its composition (67). The same team recently reported that spliced HAC1 regulates the expression of different genes according to either lipid bilayer stress or unfolded protein stress (68). In human, IRE1 TMD has also been documented for lipid-sensing properties with IRE1-detecting lipid bilayer stress to trigger UPR activation (69, 70). Moreover, by using fluorescence complementation assays, Cho and colleagues showed in cells that lipid saturation of the ER membrane with palmitate induces IRE1 (lacking NLD) dimerization through the TMD, by a mechanism involving W457 and S450 residues (71). In addition, the IRE1 TMD has also been proposed to be involved in the interaction with the membrane-embedded translocon component Sec61β. Indeed, alteration of IRE1 TMD by replacing it with calnexin TMD abolished its interaction with the translocon (72).

### IRE1 CD

The CD of IRE1 is the signaling region of IRE1. As such, it shows two main domains exerting, respectively a serine/threonine kinase activity and an RNase activity. The former leads to IRE1 transautophoshorylation upon oligomerization, thereby leading to the activation of the latter. IRE1 RNase induces the noncanonical splicing of HAC1 mRNA in yeast and of XBP1 mRNA in human, with further ligation by the Rlg1 (yeast) or RtcB (human) RNA ligases, respectively. This process allows for the synthesis of the spliced form of the XBP1s/Hac1^i^p transcription factors, thereby promoting the expression of UPR target genes (28, 73, 74). IRE1 cytosolic regions presents two main fragments: a kinase domain (yeast 674–980; human 571–832), and a RNase domain (yeast 983–1115; human 835–963) (Fig. 2*A*). Structural evidence in yeast showed that the phosphorylated IRE1 kinase/RNase domain is able to dimerize in its active form (75). This dimer is arranged in a back-to-back orientation, meaning that the kinase domain of each monomer is not in contact with their target, thus suggesting that the phosphorylation occurs by contact with another dimer (Fig. 3*E*). This activating IRE1 transautophosphorylation is further supported by the ability of the CD to form higher order oligomers (76). The phosphorylation occurring on S840, S841, and T844 in yeast IRE1 leads to the formation of both intramolecular and intermolecular (with another IRE1) salt bridges promoting the self-activation of the RNase domain (76). In contrast, hyperphosphorylation of a 28 amino acid loop (residues 864–892) containing seven serine and two threonine residues may participate in the deactivation of IRE1 by promoting its disassembly, a regulatory mechanism to adapt to a sustained ER stress (77). In human, the crystal structure of the IRE1 CD was found to resemble that of the yeast IRE1, with a back-to-back orientation bringing the RNase domains in close proximity, compatible with the cleavage of known substrates (78, 79) (Fig. 3*F*, left). This would be relevant regarding the modest conservation of sequence (∼60% similarity) (Fig. 2*B*). Although, another structure was obtained after IRE1 dephosphorylation, called the face-to-face conformation (Fig. 3*F*, right), where the RNase domains of two monomers have minimal interactions. This last orientation brings another possibility regarding the transautophosphorylation, where the kinase domain of a monomer is in close contact with the activation loop of a monomer to achieve the reciprocal phosphorylation at S724, S726, and S729 (80, 81). Despite the lake of observation of this crystal structure in yeast, the interface between the two kinase domains is conserved, suggesting that such conformation exists for all IRE1 homologs. These two states are part of a model in which IRE1 first acquires the face-to-face conformation and then proceeds to the transphosphorylation of the activation loop, leading to reorganization in a back-to-back conformation that brings two RNase domains in proximity, thus promoting IRE1 activity. Additionally, an oligomeric structure of yeast IRE1 CDs was obtained by crystallography, displaying fourteen monomers assembled in seven back-to-back dimers exerting a 52 degree rotation between each dimer, therefore confirming the ability of the CD to form high order oligomer (Fig. 3*G*) (76). Nevertheless, this structure does not take into consideration the constraints imposed by the membrane insertion of IRE1. A foci structure containing five IRE1 dimers was further observed in yeast by microscopy. Altogether, it shows that IRE1 monomers gather into larger signaling platform at the ER (63). To allow such a complex, the IRE1 linker sequence positioned between the kinase/RNase and the TMDs might be of importance by providing flexibility to the protein. In agreement with this, an *in vitro* experiment evaluating the oligomerization capacity of the cytosolic portion of IRE1 showed that deletion of the linker region prevents formation of oligomers (76). To date, there is no available crystal structure of a full-length IRE1 in any species, thereby preventing the elucidation of the exact conformation of IRE1 active form, since its oligomerization and activation may also be governed (at least in part) by the luminal domain.

While IRE1 activation might be subjected to different modes that remain debated (see Activation in ER lumen below), it is possible that the changes in sequence and structure between yeast and human IRE1 (Figs. 2*A* and 3, *A* and *B*) lead to modulation of the signaling properties. Especially, as the luminal domain is the most divergent between species, it can confer a variability in binding misfolded proteins or other partners, leading to the complexification and fine-tuning of this signaling. This would be concomitant with the appearance of other UPR sensors in metazoans. Nonetheless, one must notice that despite the three UPR sensors are expressed in *Caenorhabditis elegans*, the IRE1–XBP1s axis was shown to represent the main path to cope with ER stress in this organism (82). Additionally, while the CD retains a similar structure between species, the changes in sequence and length of this domain (∼50 amino acids more in the yeast IRE1 compared to human) may modify the oligomerization kinetic as yeast IRE1 was observed to form higher order oligomer, which were not observed in human so far. Those differences also emerge as a parameter regulating the binding and response to small molecules. Indeed, quercetin, a flavonoid, was first found to bind to yeast IRE1 in a small pocket formed by kinase and RNase domain residues (amino acids 838–844 and 1036–1040, called Q-site) (83). This compound induces a modification of yeast IRE1 nucleotide-binding site and further activates IRE1. Later, an O-glycoside quercetin derivative, the quercitrin, was characterized to bind to another binding site, located at the dimer interface region of the human RNase domain (amino acids 830–834). This molecule leads to an opposite effect, by inhibiting IRE1 (84). Altogether, it suggests that IRE1 from yeast and human respond differently to external stimuli.

## Protein-protein interactions regulating IRE1 signaling: the IRE1 signalosome

### Protein–protein interactions

#### Activation in ER lumen

IRE1 luminal domain has mainly been described as responsible for sensing misfolded proteins. Yet these sensing mechanisms are not fully understood and three models are currently proposed. On the one hand, the “direct association” model where peptides exposed at the surface of improperly folded proteins bind directly to IRE1 NLD, promoting its oligomerization and further activation. This model was first thought to occur in yeast but not human IRE1 (85, 86), however, a recent study described this mechanism in human where the direct binding induces a conformational change and leads to IRE1 oligomerization (87). On the other hand, the “competition models” presents IRE1 as a signal transducer (instead of direct sensor), where the chaperone BiP interacts with IRE1 NLD to repress IRE1 signaling. Here, two mechanisms were proposed and are still a matter of debate in the field. One sustains that in the absence of improperly folded proteins, the ER chaperone ERDJ4 binds IRE1 NLD and recruits BiP substrate binding domain to IRE1 NLD, stabilizing its monomeric form and inhibiting its oligomerization (88, 89). The other mechanism involves direct binding of BiP nucleotide binding domain to IRE1 NLD, where misfolded proteins bind BiP substrate binding domain, triggering an allosteric conformational change, releasing BiP from IRE1 and enabling its oligomerization (90, 91, 92). In both cases, upon presence of misfolded proteins, BiP is titrated away from IRE1, thus allowing its oligomerization and further activation. Such mechanisms do not seem to exist in *S. cerevisiae* (with the BiP ortholog kar2) (93, 94). Interestingly, a similar mechanism seems to occur for IREβ with the ER chaperone AGR2, where the last binds to IREβ luminal domain to favorize the monomeric state (51, 95).

In addition to those models, other PPis in the ER lumen were shown to modulate IRE1 activation. Immunoprecipitation coupled to mass spectrometry experiments, followed by biochemical approaches confirmation, showed that the ER chaperone HSP47 interacts with IRE1 NLD with high affinity upon ER stress, competing with BiP binding and leading to IRE1 oligomerization or stabilization of the oligomeric state (96). At last, oxidation of C148 in IRE1 luminal domain allows the protein disulfide isomerase PDIA6 to form disulfide bonds with oligomeric IRE1 and converting it into monomeric entities therefore limiting its signaling (97, 98). ER-stress induced phosphorylation of another protein disulfide isomerase, PDIA1, was also reported to interact with IRE1 NLD, leading to an attenuation of excessive IRE1 activation (99).

#### Oligomerization

IRE1 oligomerization is necessary to ensure signaling. IRE1 can dimerize and possibly form high order oligomers composed of several dimers. However, it remains unclear how the degree of oligomerization defines IRE1 signaling outputs. While IRE1 tetramers are thought to preferentially catalyze XBP1 splicing, IRE1 dimers tend to be more prone to mediate RIDD (55, 76). Using single molecule tracking and HaloTag, IRE1 was shown to mainly exist in an inactive back-to-back dimer (100). Upon ER stress, IRE1 dimers form transient higher order oligomers, allowing the reciprocal dimers to be positioned in a “face-to-face like” configuration. Therefore, it is interesting to focus on interactions able to fine tune IRE1 oligomerization state. In this regard, studies reported that the translocon subunit Sec61β interacts with IRE1 TMD, forming a complex with another translocon subunit Sec63, preventing IRE1 oligomerization and further activation by recruiting BiP (72, 101, 102). Additionally, it was shown that the ribosome-associated complex (RAC) chaperone is involved in the regulation of IRE1 oligomerization state. Observation of IRE1 cluster foci using microscopy revealed that RAC promotes IRE1 high order oligomerization to enhance XBP1 mRNA splicing but not RIDD (103). However, the physical interaction between RAC and IRE1 needs further confirmation. The Abelson tyrosine-protein kinase 1 (c-ABL) was found to interact with IRE1 CD upon ER stress. Independently of its kinase activity, c-ABL acts as a scaffold protein and promotes IRE1 oligomerization and further hyperactivation, leading to RIDD and terminal UPR (104). Moreover, c-ABL modulates the IRE1 signaling cascade by phosphorylating the tRNA ligase RtcB at least on three tyrosine residues (306, 316, 475), with Y306 phosphorylation reducing RtcB interaction with IRE1 and subsequently XBP1 mRNA splicing (105). One might also hypothesize that dissociation of the IRE1–RtcB complex by c-ABL might leads to the formation of IRE1 clusters able to perform RIDD. Altogether, it confers a role of switch to c-ABL, able to fine-tune IRE1 signaling through different mechanisms. At last, a link between IRE1 signaling and cytoskeleton was unveiled. The nonmuscle myosin IIB (NMIIB) interacts with IRE1 during ER stress. While it seems that NMIIB is not directly involved in IRE1 phosphorylation and dimerization, NMIIB drives IRE1 foci formation, promoting splicing of XBP1 mRNA (106). Interestingly, this mechanism was found to require the ATPase activity of NMIIB as well as a functional actin network, suggesting that the actomyosin contractility of NMIIB is required for IRE1α signaling (106).

#### RNase activity

Regarding interactions directly regulating the RNase activity, the previously described relationship between IRE1 and c-ABL was also found to act as a genotoxic stress sensor, by promoting RIDD activation and further cleavage of PPP2R1A and RuvB-like AAA ATPase 1 mRNA, two proteins involved in dephosphorylation of DNA damage proteins (107). The apoptosis regulator BAX (BAX) and Bcl-2 Homologous Antagonist/Killer (BAK), two proapoptotic proteins (108), were shown by coimmunoprecipitation to bind IRE1 (preferentially in its active form) upon high ER stress and were found to promote XBP1 mRNA splicing (109). Similarly, other PPis were found to enhance IRE1 signaling. This is illustrated by the association of IRE1 with the Bcl-2 family members Bcl-2-like protein 11 (BIM) and PUMA, leading to sustained XBP1 mRNA splicing upon ER stress (110).

Conversely, some PPis were shown to impair IRE1 RNase activity. An IRE1 immunoprecipitation coupled to mass spectrometry experiment identified RNH1, a ribonuclease inhibitor, to be present in complex with IRE1. This result was confirmed using both immunoprecipitation and proximity ligation. Surprisingly, ER stress increases this interaction between IRE1 and RNH1, thereby reducing XBP1 mRNA splicing (111). This counterintuitive outcome might indicate that IRE1–RNH1 interaction acts as a regulatory mechanism, where RNH1 decreases IRE1 activity upon prolonged ER stress. The BAX inhibitor-1 (BI-1) was found to compete with BAX and BAK for IRE1. BI-1 interacts with the IRE1 C-terminal region and reduces IRE1 RNase activity (112, 113). Under basal conditions, the interaction of the SH2/SH3-containing adaptor protein 1 with IRE1 carboxy-terminal region was shown to repress IRE1, a mechanism proposed to avoid a signaling leakage (114).

### Protein modification

#### Phosphorylation-dependent regulations of IRE1 signaling

Evidences unveiled a role of IRE1 phosphorylation in determining its signaling outcomes (115). As such, IRE1 faces phosphorylation-dephosphorylation cycles relying on PPis. These regulating events were studied and revealed interesting mechanisms. Regarding the downregulation of IRE1 signaling through dephosphorylation, two yeast phosphatases—Ptc2p serine/threonine phosphatase of type 2C and Dcr2—were found to interact with phosphorylated IRE1 and further dephosphorylate it to downregulate the splicing of HAC1 mRNA (26, 116). Interestingly, it was found that Dcr2 specifically interacts with IRE1 when phosphorylated at S840 and S841 residues (116). This finding was extended to human thanks to another study describing IRE1 interaction with the ER membrane phosphatase PP2Ce, the human homolog of the *S. cerevisiae* Ptcp2 (117). Here, PP2Ce dephosphorylates IRE1 S724 upon ER stress, acting as a negative regulator of IRE1 signaling. Of note, both IRE1α and IRE1β were shown to partner with PP2Ce, unveiling a probable common regulatory mechanism (117). Coimmunoprecipitation assays found that the scaffold protein RACK1 interacts with IRE1 linker domain (between the kinase and the TMD) (118, 119). In basal condition, RACK1 recruits the phosphatase PP2A, which dephosphorylate IRE1 (120). A recent article uncovered through screening different serine-threonine phosphatases that the small carboxy-terminal domain phosphatase 3 was able to interact directly with IRE1 and further dephosphorylate the protein (120). We previously mentioned the crosstalk between the UPR sensors. In this regard, a study investigated the role of the PERK pathway in PPi-dependent attenuation of IRE1 signaling. Upon acute ER stress, PERK-prolonged activation leads to the phosphatase RPAP2 to interact and dephosphorylate IRE1, thereby decreasing its signaling (41). Another mechanism involving the protein tyrosine phosphatase PTP1B was shown to regulate both catalytic and scaffolding functions of IRE1 (105, 121). Here, PTP1B deficiency or catalytically inactive impairs XBP1 splicing and c-Jun-N-terminal-kinase (JNK) activation (121), while further investigations revealed that PTP1B dephosphorylates RtcB, thus enhancing its association with IRE1 for XBP1 mRNA splicing (105). However, to date, direct interaction between IRE1 and PTP1B requires confirmation.

Conversely, other proteins were found to phosphorylate IRE1. Interestingly, the previously mentioned IRE1–RACK1 complex was found to play another role. Upon increased ER stress, RACK1 recruits the AMP-activated protein kinase, which phosphorylates IRE1 (118, 119). Hence, RACK1 acts as a platform regulating IRE1 signaling according to ER stress through the phosphorylation status. Glucagon-stimulated PKA was reported to phosphorylate IRE1, indicating a role in glucose metabolism and suggesting an ER-lumen–independent activation (122). At last, a mechanism by which KRas mutated tumors elaborate tumor resistance to KRas inhibitors was shown to involve ERK1/2-mediated phosphorylation of IRE1, thus preventing its degradation by the ERAD machinery (120). This led us to further document the role of posttranslational modifications (PTMs) of IRE1 and its degradation.

#### PTMs and IRE1 degradation

N-glycosylation is a PTM occurring during synthesis and maturation of ER proteins, participating in their folding and functionality (8, 123). IRE1 is N-glycosylated at N176 (124, 125) but there is no clear evidence mentioning a role for this PTM in regulating IRE1 activity. Despite that, few studies reported that mutations preventing this PTM do not impair IRE1 folding nor oligomerization properties (124, 125). Also, N-glycosylation disruption causes ER stress, and some might hypothesize that it could be a sensor-initiating IRE1 cascade.

Alternatively, ERAD was shown to control IRE1 expression and activity, as shown by the SYVN1/SEL1L-dependent ubiquitination of IRE1, promoting its degradation (126, 127). In this case, IRE1 is sent to ERAD in basal conditions, where both BiP and the lectin protein OS9 interacts with IRE1 in the lumen, recruiting the SYNV1/SEL1L complex and subsequently allowing IRE1 cytosolic ubiquitination by the E3 ligase SYNV1 (127). OS9 being a lectin, we suggest that it recognizes IRE1 through its N-glycan chain, therefore conferring the N-glycan a role in IRE1 stability. Cytomegalovirus M50 was found to take advantage of this ERAD-dependent IRE1 degradation, where the M50 viral protein interacts and tethers with both IRE1 and SEL1L to promote IRE1 degradation (128). IRE1 was also found to be a substrate of the E3 ubiquitin-protein ligase CHIP (also involved in ERAD). Authors proposed a dual role for CHIP-dependent IRE1 ubiquitination during ER stress: first, ubiquitination of IRE1 K545, enhancing IRE1 phosphorylation and second, of IRE1 K828, facilitating recruitment of TNF receptor-associated factor 2 (TRAF2) for JNK pathway activation (129). This is supported by another study, where the E3 ligase RNF13 interacts with IRE1, exerting enhanced XBP1 splicing and JNK activation (130). On another note, a recent study identified IRE1 to be a substrate of the mitochondria ubiquitin ligase MARCHF5 (MITOL), which ubiquitinylate IRE1 K481 with a K63 ubiquitin chain at mitochondria-ER contact sites (131). This ubiquitination was proposed to disrupt BIM stabilizing interaction with IRE1 oligomers, thus inhibiting IRE1 signaling (110, 131).

## IRE1 PPis associated with other functions/outcomes

### Protein–protein interactions

#### Apoptosis

IRE1 signaling has been connected to the activation of the apoptosis cascade. Indeed, upon prolonged ER stress, IRE1 RIDD activity cleaves nonselectively several prosurvival miRNAs. Among them, miR-17, a miRNA known to repress TXNIP expression, subsequently triggers caspase 1 and 2/interleukin-1β–dependent apoptosis (36, 132). Of note another report showed that ER stress did not lead to the activation of caspase 2 to initiate apoptosis (133). Several other studies have also connected IRE1 PPis with cell death, occurring during terminal UPR (134). This is linked to the activation of the proapoptotic JNK pathway, which first involves the interaction of IRE1 with TRAF2 and the further recruitment ASK1, also known as apoptotic signaling kinase 1 (135, 136). ASK1 may then phosphorylate JNK, thereby initiating the JNK pathway and leading to apoptosis. A new actor in the TRAF2/ASK1/JNK axis was also identified as the ASK1-interacting-protein 1 (AIP1), which, upon ER stress, is recruited on IRE1 prior to TRAF2 and further promotes JNK pathway activation (137). Additionally, this study presented evidence that AIP1 could enhance IRE1 dimerization (137). We previously mentioned IRE1 interactions with the Bcl-2 family member BIM and PUMA. These interactions occur through the BH3 domain of these proteins, which is necessary for their canonical proapoptotic signaling (109). It was shown that knock out of BIM and PUMA reduces both IRE1 RIDD and JNK activation (110). Therefore, BIM and PUMA might promote apoptosis by sustaining cleavage of prosurvival miRNAs by RIDD and activation of the JNK pathway. Accordingly, the other members of the Bcl2-2 family BAX and BAK, also containing BH3 domains, may act in a similar manner by interacting with IRE1 (109). Therefore, it confers a noncanonical role of these BH3 domain proteins in inducing cell death. Conversely, we also hypothesize on this model that the previously described protein BI-1 can inhibit cell death by competing with BAX and BAK interactions with IRE1. More recently, another IRE1 PPi was reported to exert antiapoptotic functions, involving the previously described interaction between IRE1 and MITOL, shown to inhibit JNK phosphorylation (131).

#### Cytoskeleton

In the past years, few studies reported a crosstalk between UPR and cytoskeleton as mentioned with NMIIB or through the interaction of the actin-binding protein FLNA with PERK (106, 138). Interestingly, a mechanism involving both IRE1 and FLNA showed that IRE1 promotes cell migration through FLNA phosphorylation. In this study, IRE1 associates with FLNA and acts as a recruiting platform for the kinase PKCα, enabling the phosphorylation of FLNA, which in turns drives actin cytoskeleton remodeling and cell migration (139). This interaction was shown to participate in migration of cells infected by toxoplasma, where the parasite engages the IRE1–FLNA axis (140). Additionally, several high-throughput interactome screenings identified other cytoskeletal proteins such as CORO1 proteins or tubulin α and β, however, they require further validation (111, 139). IRE1 interacting with cytoskeleton proteins may also participate in membrane contact sites between ER and plasma membrane (ER-PM contact sites). This complex is known to regulate Ca^2+^ and lipid homeostasis, two processes associated with IRE1 signaling (141, 142).

#### Translation machinery: signal recognition particle and ribosome

High translational load can result in nonefficient protein folding in the ER by overwhelming the folding capacity of this compartment, hence, triggering ER stress. Given the proximity of IRE1 with the protein synthesis machinery, a crosstalk has been discovered between IRE1 signaling and mRNA translation. Cross-linking experiments revealed interactions between IRE1 and translating ribosomes at the cytosolic ER surface, through the ribosomal subunits RPL9, RPL10A, RPS14, and RPS24 (143). In the same study, immunoprecipitation analyses showed IRE1 interaction with proteins from the signal recognition particle (SRP) (143). These results correlate with studies describing that XBP1 mRNA begins translation before being brought to the ER membrane by the SRP pathway to undergo IRE1-dependent splicing (144, 145, 146). Moreover, we can hypothesize a role for IRE1 in monitoring the newly synthesized polypeptides entering the ER during the cotranslational translocation, as a way to avoid protein accumulation into the ER. Of note, those interactions with SRP and ribosomal proteins did not modify the catalytic functions of IRE1, thus suggesting a catalytic-independent regulation of the UPR which remains to be further investigated.

#### Calcium homeostasis

The ER is an intracellular organelle known to be involved in the maintenance of Ca^2+^ homeostasis. It was suggested that IRE1 could play a role in regulating Ca^2+^ metabolism independently of its enzymatic activities (147, 148). Indeed, IRE1 CD was shown to interact with the inositol-1,4,5-trisphosphate receptors. This PPi allows mitochondria to localize close to the ER in the mitochondria-associated ER membrane, another membrane contact site, and further promote calcium transfer to support mitochondrial respiration (148). This is supported by recent findings describing the interaction between IRE1 and the stromal interaction molecule 1 (STIM1) in the ER lumen (149). This interaction connects the UPR and Ca^2+^ metabolism, where STIM1 promotes XBP1 mRNA splicing upon ER Ca^2+^ store depletion and IRE1 promotes STIM1-dependent ER-PM contact sites, triggering the store operated Ca^2+^ entry pathway.

### Direct IRE1 kinase–dependent phosphorylation

For many years, the only known substrate of the IRE1 kinase domain was IRE1 itself. However, few recent publications reported novel targets for IRE1 kinase activity. The previously described IRE1–RACK1 axis was shown to participate in BCL-2 phosphorylation by IRE1 at S70, a phosphorylation site known to exert antiapoptotic functions (119). Liu and colleagues also showed that IRE1 interacts and promotes AMP-activated protein kinase phosphorylation exerting prosurvival signaling, nevertheless the direct/indirect modification remains unclear (119). The fragile X mental retardation protein (FMRP) was also identified as a new IRE1 kinase substrate, possibly regulating cholesterol transport and efferocytosis (150). In addition, pumilio, a RNA-binding protein in *drosophila*, was shown to be phosphorylated by IRE1, allowing its binding to XBP1 mRNA and protecting it against IRE1 RIDD activity (151). PUM1, the human homolog of pumilio, has also been shown to be phosphorylated by IRE1 (151). Another study has reported an interaction between IRE1 and the IκBα kinase complex (IKK). The use of a kinase defective IRE1 mutant (K599A) revealed a reduced phosphorylation of IκBα kinase, rescued by transfection with a WT IRE1 construct (152). This links IRE1 with the NF-κB pathway, involved in many cellular processes such as inflammation or apoptosis. At last, the sphingosine 1-phosphate lyase (SPL) was shown to be phosphorylated by IRE1, inhibiting its enzymatic activity and resulting in activation of a mitochondrial-dependent UPR (153). Even though the experiments performed were mostly *in vitro*, these discoveries open a new field in the exploration of IRE1 kinase–dependent signaling and raise questions about potential implication of IRE1 in processes not limited to the UPR.

## Concluding remarks and outstanding questions

**Figure 4:**
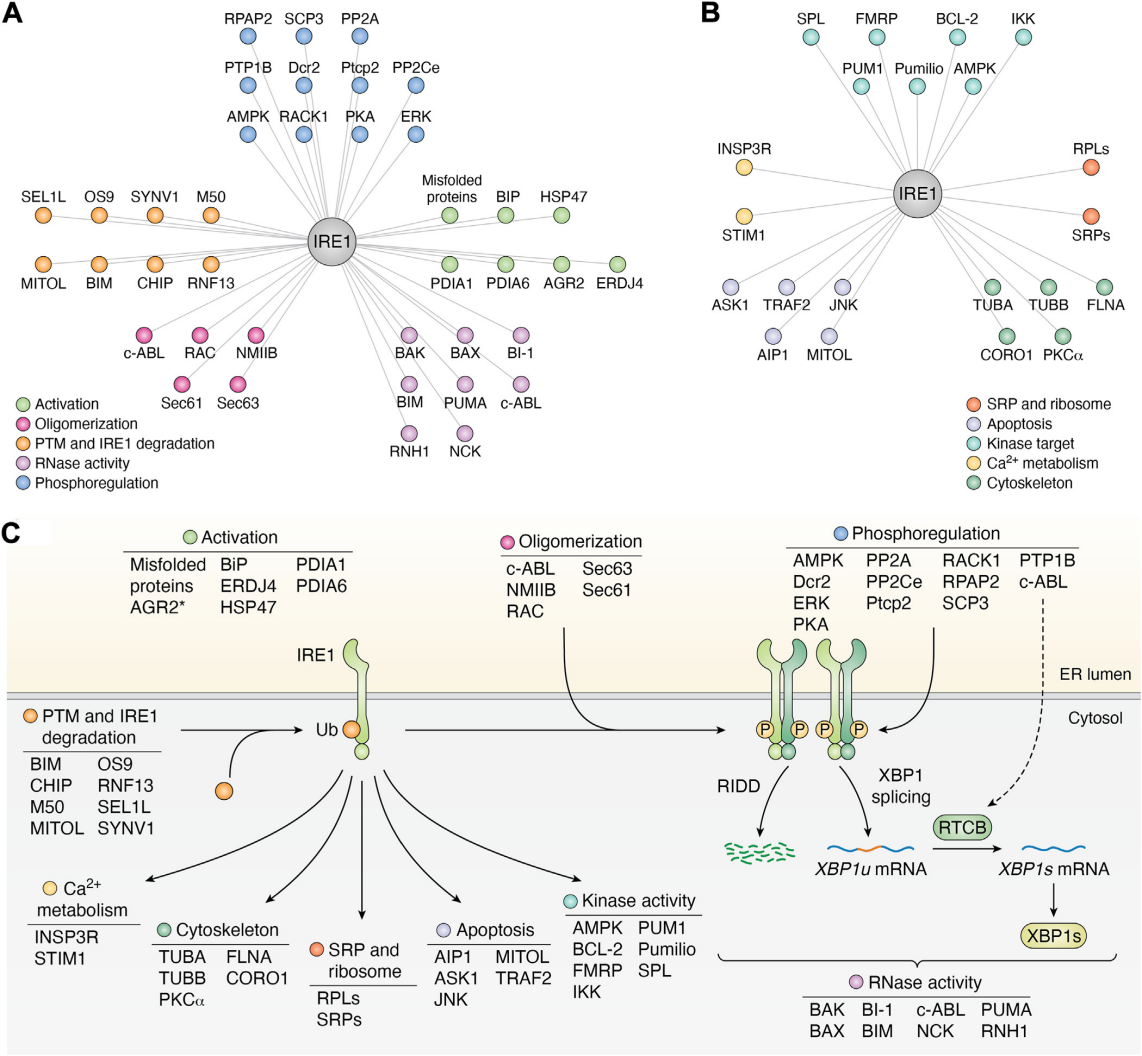
**Summary of the current IRE1 interactome.** Clusters of IRE1 interactors regulating IRE1 signaling (*A*) or exerting noncanonical functions (*B*). *C*, integrated view of IRE1 interactions mediating its signaling and other functions. (∗) indicates IRE1β-specific interactions. AIP1, ASK1-interacting protein 1; FMRP, fragile X mental retardation protein; IKK, IκBα kinase complex; IRE1, inositol-requiring enzyme 1; MITOL, mitochondria ubiquitin ligase MARCHF5; SPL, sphingosine 1-phosphate lyase

IRE1 first appeared as a transducer of the UPR, in order to restore a balance in ER homeostasis and help cells adapt to stress. The evolutive interactome unveils possible new roles of IRE1 in mammals. IRE1 is connected with immunity, inflammation, and metabolism and is therefore involved in disease progression (154, 155). While most of the studies focus on IRE1 RNase activity, understanding the dynamic of IRE1 interactions may also provide useful insights on disease development and potential therapies. This review summarizes the known IRE1 interactors and classifies them according to their role in regulating IRE1 signaling (Fig. 4*A*) or exerting noncanonical (RNase independent) functions (Fig. 4*B*). While a majority of the interactors were characterized to regulate IRE1 canonical signaling, an increasing number of evidences tend to attribute to IRE1 a wide range of diverse functions (Fig. 4*C*).

IRE1 signaling can therefore be regulated by interactors at every step of its activation. We term this protein interaction network regulating IRE1 signaling functions as the IRE1 signalosome. The latter can be modulated by specific signals; hence, deciphering how the regulation of the IRE1 cascade exerts different cellular outcomes might represent an appealing avenue to better understand IRE1 signaling. We found recent studies describing new targets of the IRE1 kinase activity. Protein phosphorylation is a crucial mechanism in transducing signals and regulating cellular functions (156). Considering IRE1 as a regular kinase opens a new field of investigations. Studying the sequence and modeling the complex of the known IRE1-dependent phosphorylation could provide some insights on potential other targets. Lastly, IRE1 is known for its role in regulating the translational load through RIDD in the cytosol. However, some studies reported a possible localization of IRE1 in the nucleus (143, 157, 158, 159) and confirming this would require further investigations. Considering the continuity between ER membrane and nuclear envelope, we can hypothesize a nonconventional localization of IRE1, where it would unveil a new landscape of regulators and regulate gene expression independently from RIDD.

## Conflict of interest

E. C. is the founder of Thabor Therapeutics (https://www.thabor-tx.com/). The other authors declare that they have no conflicts of interest with the contents of this article.
